# HIV-1 viraemia and drug resistance amongst female sex workers in Soweto, South Africa: A cross sectional study

**DOI:** 10.1371/journal.pone.0188606

**Published:** 2017-12-15

**Authors:** Jenny Coetzee, Gillian Hunt, Maya Jaffer, Kennedy Otwombe, Lesley Scott, Asiashu Bongwe, Johanna Ledwaba, Sephonono Molema, Rachel Jewkes, Glenda E. Gray

**Affiliations:** 1 Perinatal HIV Research Unit (PHRU), Faculty of Health Sciences, University of the Witwatersrand, Johannesburg, South Africa; 2 School of Public Health, University of the Witwatersrand, Johannesburg, South Africa; 3 National Institute for Communicable Diseases (NICD), Sandringham, South Africa; 4 Department of Molecular Medicine and Haematology, Faculty of Health Science, School of Pathology, University of the Witwatersrand, Johannesburg, South Africa; 5 Gender and Health Research Unit, South African Medical Research Council, Pretoria, South Africa; 6 Office of the President, South African Medical Research Council, Cape Town, South Africa; University of Pittsburgh, UNITED STATES

## Abstract

**Background:**

HIV drug resistance (HIVDR) poses a threat to future antiretroviral therapy success. Monitoring HIVDR patterns is of particular importance in populations such as sex workers (SWs), where documented HIV prevalence is between 34–89%, and in countries with limited therapeutic options. Currently in South Africa, there is a dearth in evidence and no ongoing surveillance of HIVDR amongst sex work populations. This study aims to describe the prevalence of HIVDR amongst a sample of female sex workers (FSWs) from Soweto, South Africa.

**Methodology:**

A cross-sectional, respondent driven sampling (RDS) recruitment methodology was used to enrol FSWs based in Soweto. Participants were tested for HIV and undertook a survey that included HIV knowledge and treatment status. Whole blood specimens were collected from HIV positive FSWs to measure for CD4 counts, viral load (VL) and perform HIVDR genotyping. Frequencies were determined for categorical variables and medians and interquartile ranges (IQR) for the continuous.

**Results:**

Of the 508 enrolled participants, 55% (n = 280) were HIV positive and of median age 32 (IQR: 20–51) years. Among the HIV positive, 51.8% (132/269) were defined as virologically suppressed (VL < 400 copies/ml). Of the 119 individuals with unsuppressed viral loads who were successfully genotyped for resistance testing 37.8% (45/119) had detectable drug resistance. In this group, HIVDR mutations were found amongst 73.7% (14/19) of individuals on treatment, 27.4% (26/95) of individuals who were treatment naïve, and 100% (5/5) of defaulters. One phylogenetic cluster was found amongst treatment naïve FSWs. The K103N mutation was detected most commonly in 68.9% (31/45) individuals with HIVDR mutations, with 20/26 (76.9%) of treatment naïve FSW with detectable resistance having this mutation. The M184V mutation was found in both FSWs on treatment (12/14, 85.7%) and those defaulting (1/5, 20.0%).

**Discussion:**

More than one third (45/119) of the genotyped sample had HIVDR, with resistance to the NNRTI class being the most common. Almost half of HIV positive FSWs had unsuppressed viral loads, increasing the likelihood for onward transmission of HIV. Disturbingly, more than 1:4 treatment naïve women with unsuppressed viral loads had HIVDR suggesting that possible sexual transmission of drug resistance is occurring in this high-risk population. Given the high burden of HIVDR in a population with a high background prevalence of HIV, it is imperative that routine monitoring of HIVDR be implemented. Understanding transmission dynamics of HIVDR in FSW and its impact on treatment success should be urgently elucidated.

## Background

HIV drug resistance (HIVDR) to first and second line antiretroviral (ART) regimens pose serious limitations to therapy success. Thus the prevention thereof has become a global priority[1]. Given the high HIV prevalence found in sex worker (SW) populations and the roll-out of the *South African National Sex Worker HIV Plan 2016–2020*[2] -which sees the provision of access to universal test and treat (UTT)—it is critical that we begin to understand HIVDR amongst SWs. This is especially important when considering the limited evidence currently available on HIVDR amongst SWs in South Africa. In the event that a significant proportion of the population develop, transmit or acquire HIVDR, the gains made in treating and preventing HIV in recent years would potentially be lost [3].

South Africa has the largest ART program in the world, with 3 million people initiated during 2015. The country is also scaling up service delivery to SWs as a means to curb the epidemic [2]. While survival, quality of life, and prevention are improved through such efforts, the risk of HIV drug resistance (HIVDR) mutations increases as more people are placed on ART[3, 4]. Longer lifetimes on ART allows for more time for resistance to develop, and pressure on the public health system may lead to events such as drug-stock outs, in turn leading to treatment interruptions [5]. Compounded by the infrequency of viral load (VL) monitoring and that appropriate action in the face of virologic failure may not always be taken, patients may continue on failing regimens, with an accumulation of HIVDR mutations developing [3]. An accumulation of drug resistant mutations may be a risk even when adherence results in viral suppression, due to multiple infections and the additional sexual acquisition of transmitted drug resistance (TDR). In South Africa, provision is largely made for HIVDR testing as a prerequisite to initiating a third line regimen. At present HIVDR testing is usually available through centralised processes in government clinics and hospitals in Southern Africa–primarily because of issues around laboratory capacity and because the results may be poorly understood by non-specialist clinicians [6].Data from the WHO suggests that between 2013–2014 in South Africa, while 100% of clinics were reported to prescribe ART, retention in care was 67.8% and only 27.2% of patients collected their ART pills on time, both early warning signs for the development of HIVDR[3].

The WHO’s 2012 drug resistance report highlights the potential of increasing HIV drug resistance–both acquired drug resistance (ADR) which is a result of drug selection pressure, and TDR, when an individual with no exposure to ART is infected with virus that has existing drug-resistant mutations [5]. A meta-analysis of data from South Africa during 2000–2010 did not find any increase in TDR, and suggested that South Africa falls into what the WHO classifies as a “low prevalence” country for TDR, with less than 5% TDR[7]. However, more recent data suggests that drug resistance is indeed increasing. In low-middle income countries, the WHO reported levels of HIVDR to increase between 2004–2014, with higher levels of TDR being seen amongst treatment naïve individuals, particularly to non-nucleoside reverse transcriptase inhibitors (NNRTIs), currently used in many first-line ART regimens [5]. In South Africa, data from a NICD study released in 2016 reported TDR at 14% [3, 8]. Modelling estimates of the South African epidemic suggest that by 2032, should a test and treat strategy result in ART initiation irrespective of CD4 count, with no other changes to diagnosis and retention rates, HIVDR to NNRTIs will be present amongst 32% of the total HIV positive population, and that 30% of these (9.6% of the total HIV positive population) will also have a VL ≥500 copies/ml meaning they are infectious [9].

Moderate levels of HIVDR (9%, 25/277) was found amongst pre-treatment patients from a sample of clinics across South Africa between 2013–2014 [10]. In addition, evidence from KwaZulu Natal found that amongst adults who had recently been diagnosed (≤ 24 months), 7.1% had some form of resistant mutation, with the most common NNRTI mutation being K103N (27, 3.8%). NRTI resistant mutations were detected in 10 (1.4%) adults with 8 having only a single NRTI mutation[11]. A 24-week prospective study of treatment naïve HIV positive individuals in Uganda found that treatment interruptions >48 hours could result in drug resistance (13%) [12].

Extremely high HIV prevalence has been documented amongst female sex workers (FSWs) in South Africa. The 2013 *South African Health Monitoring Survey* (SAHMS) found levels in Johannesburg, Durban and Cape Town of 71.6%, 53.5%, and 39.7% [13]. The study also found that knowledge of HIV status was high amongst female sex workers (FSWs), yet uptake of treatment was poor (23.4–45.3%). In Johannesburg for example, 38% of FSWs knew their status but were treatment naive, of HIV positive FSWs, 21.6% were unaware of their HIV status, and only 12.2% of known positive FSWs were on ART [13]. A cross sectional survey of women selling sex along a major trucking route, showed an HIV prevalence of 89.9% [14]. The sample presented within this paper had an adjusted HIV prevalence of 53.1% for FSWs in Soweto[15]. Sex worker vulnerability to HIV is driven by several factors including sexually risky behavior and violence[13, 15, 16]. Evidence has shown men who purchase sex are more like to engage in sexually risky behaviors such as violence and poor condom uptake [17–19]. Violent men are also more likely to be HIV positive[17], and violence is significantly associated with treatment defaulting [20] which is a key factor in developing HIVDR. Together, these highlight the possible vulnerability of FSWs to HIVDR. In addition, recreational drugs with a combination of marijuana, heroin and ARVs are growing in popularity in South African townships, yet nothing is known about its impact on HIVDR.

Regular surveillance for HIVDR in key affected groups such as SWs, is not routinely undertaken in South Africa, and available evidence globally is based upon very small samples from only a few locations. Amongst SW populations within Kenya, evidence suggests a 22% prevalence of HIVDR [21]. An unpublished Kenyan study suggested that 8.3% of ARV treated FSWs within the Pumwani cohort had HIVDR (5/60)[22]. Studies of SWs in Argentina have found HIVDR in 12/62 (19%) [23], and 3/16 (18.8%) [24]. A 2005 study of FSWs and injecting drug users (n = 11) along the US-Mexico border found two out of three drug naïve FSWs to have low-level resistance mutations. In addressing the dearth of data on HIVDR amongst the sex work population, this study aims to describe the prevalence of HIVDR mutations amongst a sample of FSWs from Soweto, South Africa.

## Methodology

We present an analysis from a convenience sample of HIV positive FSWs from a larger cross-sectional, respondent driven sampling survey. We previously reported the overarching methodology, baseline characteristics and population-level prevalence of HIV in the overarching study [15]. The study was nested within an existing sex work program at the Perinatal HIV Research Unit in Soweto, South Africa. The surrounding township is on the outskirts of Southern Johannesburg, predominantly urban and peri-urban, low-income with limited educational and employment opportunities. It comprises >40 suburbs within 61km^2^, is estimated to house >2 million inhabitants and has > 3,000 drinking establishments. The sex work program fixed clinic was used as a base for this study. Respondent driven sampling (RDS) recruitment was used in line with the guidelines provided by the World Health Organisation[25]. This method is a popular way to recruit marginalised populations [26, 27].

Recruitment was undertaken between February-September 2016 (n = 508). Inclusion criteria for the present study was enrollment into the larger study (biologically female, >18 years, currently engaged in the sale of sexual services in Soweto, knew their recruiter, and gave voluntary informed consent to participate in both the survey and HIV testing), and tested HIV positive. A VL must have been successfully undertaken, and if VL ≥500 copies/ml then HIVDR genotyping was performed. To avoid duplicate enrollment, fingerprints (left, index finger) were scanned and an algorithm recorded and compared for every enrollment using the FingerTec OFIS system (http://www.fingertec.com/images/brochure/Ofis-E.pdf).

After screening and written consent, participants completed a 45-minute, interviewer-administered questionnaire and then underwent HIV counselling and testing. Using the WHO guidelines, two rapid tests were conducted[28]. HIV positive participants underwent a venous blood draw for CD4, VL and HIVDR genotyping. Participants were provided with a maximum of three uniquely coded coupons to refer other FSWs to the study and reimbursed R100.00 ($7.69), later increased to R120.00 ($9.25) due to additional costs to participate. A further R20.00 ($1.56) was paid for recruiting other participants into the study.

HIV VL testing were performed using the Abbott RealTi*m*e HIV-1 assay (Abbott Molecular, Des Plaines, Il)by Clinical Laboratory Services (CLS, Johannesburg, South Africa) within 40 minutes road transport from the clinic collection site for same day VL testing service. CD4 enumeration was conducted by the National Health Laboratory Service (NHLS) as part of standard of care, using the PanLeucogating (PLG) technology (Beckman Coulter, Miami, Fl). Drug resistance genotyping was undertaken by the National Institute of Communicable Diseases (NICD). Sequencing of the polymerase gene was performed using a validated in-house assay [29]. The procedure involved the generation of a nested PCR amplicon spanning the entire protease and p66 and p51 regions of the reverse transcriptase genes. Genotypic resistance was defined as the presence of resistance mutations associated with impaired drug susceptibility, using the Stanford University HIV Drug Resistance Database (https://hivdb.stanford.edu/). Phylogenetic analysis of nucleic sequences was performed using an HKY85 model to establish linkages using PAUP. Specimens were repeated from extraction stage to confirm result. Rates of transmission of resistance were determined by calculating point prevalence.

Survey data and HIV test results were collected during interviews directly onto Lenovo tablets into the REDCap electronic data management system[30], hosted by the University of the Witwatersrand, South Africa. The database housed built-in skip patterns and algorithms, with missing values being highlighted before an interviewer could move to the next survey section. Data could be viewed live on the REDCap database to ensure that queries were rapidly addressed before participants left the study site. The system also enabled interviewers to insert detailed comments or explanations on responses they wished to discuss. A 48-hour response-time to checking and closing queries was implemented during the study. This aided in ensuring high quality data was consistently captured. VL, CD4 and drug resistance data was captured into the system as it became available, using participant’s unique identifier to match results. Duplicate data were collected on a convenience sample of 12% of the overall sample with an error rate of 0.6%. Twenty-one CD4 results were either lost or rejected by the NHLS for reasons unrelated to the study. Ethical approval for this study was provided by the Human Research Ethics Committee (Medical) of the University of the Witwatersrand, South Africa (M150740).

### Measures

Drug resistance mutations were indicated as present or absent. All mutations were categorized to indicate NNRTI, NRTI or PI genotypes. Rates of HIVDR were analyzed according to self-report treatment history.

Participants were asked their treatment history, with questions being asked dependent on a skip pattern of having tested and taking treatment. Questions were: “Have you previously tested for HIV?” (yes/no) “What was your test result?” (I do not know, negative, positive, indeterminate), “Are you on treatment?” (never taken, on treatment always, stopped taking treatment), and “In the past week how many times have you taken your treatment?” (all 7 days, 5–6 days, 3–4 days, 1–2 days, not at all). Interviewers were trained to probe for PMTCT history, and the nurse confirmed previous ART history. A new variable was then created indicating self-report treatment history as: treatment naïve (either unknown positive and never taken treatment, or known positive but never taken treatment); treatment defaulting (known positive and stopped taking treatment or treatment taken for ≤5–6 days of the past week); on treatment (known positive, on treatment always and treatment taken all 7 days). Participants were asked about their use of Nyoape, the colloquial name for a recreational drug consisting of marijuana, heroin and ARVs, only three responses reported using the drug, one was HIV positive and treatment naïve. The measure was dropped.

### Statistical analysis

Log transformations were performed on VLs that were capped at 750,000 copies/ml. VL was evaluated in 2 ways: as a log-transformed continuous measure, and dichotomised with a cutoff of ≥400 copies/ml for viral suppression. CD4 count was assessed as a continuous measure. Frequencies were determined for categorical measures whereas continuous measures were assessed by medians and interquartile ranges. Continuous measures of the HIV positive participants stratified by a cut-off of ≥400 copies/ml were compared non-parametrically using studentized t-test. Two participants with VLs between 400–500 were classified as unsuppressed, but not genotyped and so dropped from the analysis. Data were analyzed by STATA13 [31].

## Results

As shown in Table 1, HIV tests were done on 508 FSWs, with 55.1% (280/508) being HIV positive. Viral load RNA measurements were done on 96.1% (269/280) of HIV positive samples with 49.1% (132/269) of these being ≥ 400 copies/ml and defined as unsuppressed. 90.2% (119/132) were successfully amplified and sequenced for HIVDR genotyping, and 37.8% (45/119) having evidence of HIVDR mutations. Viral load or genotyping could not be done (n = 13) due to: one refused blood draw, one failed blood draw, two viral load between 400–500, four samples were haemolysed and five missing.

**Table 1:** Summary of overall study sample.

	n (%)
HIV tests done	508
HIV positive	280 (55.1)
Tested for RNA VL	269 (96.1)
VL ≥400 copies/ml	132 (49.1)
Successfully amplified	119 (90.2%)
HIVDR Mutations	45 (37.8)

Table 2 presents summary characteristics for all HIV positive FSWs. The median age in Soweto was 32 (20–51) years, with CD4 being 473 (311–701) cells/mm^3^, and VL log_10_ being 2.4 (1.6–4.0). 51,8% (141/269) of HIV positive FSWs had a VL <400 copies/ml which in this study was defined as viral suppression. Drug resistance genotyping was only done on participants with VLs ≥400 (n = 119). The total proportion of HIV positive SWs who self-reported being on treatment was 43,9% (118/269), 50.6% (136/269) were treatment naïve, and 5.6% (15/269) were defaulting treatment. Overall one phylogenetic cluster was found comprising 2 participants.

**Table 2:** Summary characteristics of study sample overall for HIV positive and by VL using a 400 cutoff and drug resistance testing done.

	All HIV+ (n = 269)	VL ≥400 copies/ml and DR testing done (n = 119)	VL <400 copies/ml (n = 141)	p-value
Age (median yrs, IQR)	32 (20–51)	30.9 (29.7–32.1))	34.1 (33.0–35.4)	0.0001
CD4 cell count (median, IQR)	473 (311–701)	419.2(371.1–467.2)	623.8(570.2–677.3)	<0.0001
VL log_10_ copies/ml (median, IQR)	2.4(1.6–4.0)	4.2(4.0–4.3)	1.7(1.6–1.7)	n/a
*Known Positive*	220 (81.8)	85 (71.4)	127 (90.1)	<0.0001
Treatment Status				
On Treatment (n, %)	118 (43.9)	19 (16.0)	99 (70.2)	-
Treatment Naïve (n, %)	136(50.5)	95(79.8)	34 (24.1)	-
Treatment Defaulting (n, %)	15 (5.6)	5(4.2)	8 (5.7)	-
Wildtype	74 (27.5)	74 (62.2)	-	-
Detectable resistance (n, %)	45 (16.7)	45 (37.8)	-	-
NNRTI only (n, %)	29 (10.5)	29 (24.4)	-	-
NRTI only (n, %)	1 (0.4)	1 (0.9)	-	-
NNRTI+NRTI (n, %)	13 (4.7)	13(10.9)	-	-
NNRTI+NRTI+PI (n, %)	2 (0.7)	2 (1.7)	-	-
Phylogenetic clusters (n)	1 (0.4)	1 (0.9)	-	-

Also presented in Table 2 are HIV positive participants with a VL < 400 copies/ml. The median age was 34.1 years and CD4 count was 623.8 cells/mm^3^. The median VL log_10_ was 1.7 (1.6–1.7). By comparison, of FSWs with a VL ≥400 and who were successfully genotyped (n = 119), the median age was 30 years and CD4 count was 419 cells/mm^3.^ The median VL log_10_ was 4.2(4.0–4.3), 79.8% (95/119) self-reported treatment naïve, 16.0% (19/119) self-reported being on treatment, and 4.2% (5/119) were defaulting their treatment. Detectable HIVDR was found in 37.8% (45/119). NNRTI only resistance was detected in 24.2% (29/119), NRTI only amongst 0.9% (1/119), NNRTI and NRTI mutations amongst 10.9% (13/119), and NNRTI, NRTI and PI mutations amongst 1.7% (2/119).

Table 3 shows all participants successfully genotyped for HIVDR (n = 119). For those reporting being treatment naïve (n = 95), the median age was 30 (24–36) years, CD4 cell count was 381.5 (253–508) cells/mm^3^, VL log_10_ was 4.1(3.3–4.8), and 81.8% were known positives (220/269). Resistance was detected in more than a quarter (27.4%, 26/95) of treatment naïve FSWs, 24.2% (23/95) had NNRTI only mutations, 3.1% (3/95) had NNRTI and NRTI mutations, and 1 phylogenetic cluster was detected. In the same table, those reporting being on treatment (n = 19), the median age was 35 (29–40) years, CD4 count result was 285(96–530) cells/mm^3^, VL log_10_ was 4.0(3.5–4.9), and 71.4% were known positives (85/119). Resistance was detected in 73.7% (14/19) of these participants. NNRTI only resistance mutations were detected in 10.5% (2/19), NRTI only mutations in 5.3% (1/19), NNRTI and NRTI mutations in 47.4% (9/19), and NNRTI, NRTI and PI mutations in 10.5% (2/19). Finally, amongst those reporting treatment defaulting (n = 5), the median age was 33 (31–34) years, CD4 count result was 306 (195–526) cells/mm^3^, VL log_10_ was 3.9(3.9–4.1), and 90.1% were known positives (127/141). Resistance was detected in 100% (5/5) of this subset. NNRTI only was detected in 80% (4/5) and NNRTI and NRTI in 20% (1/5).

**Table 3:** Summary characteristics of HIV positive FSWs with a VL ≥400copies/ml and drug resistance testing done (n = 119), by treatment naïve, on treatment and treatment defaulting.

	Treatment naïve (n = 95)	On Treatment (n = 19)	Treatment Defaulting (n = 5)
Age (median, IQR)	30 (24–36)	35(29–40)	33 (31–34)
CD4 cell count (median, IQR)	381.5 (253–508)	284.5(96–530)	306 (195–526)
VL log_10_, copies/ml (median, IQR)	4.1(3.3–4.8)	4.0(3.5–4.9)	3.9(3.9–4.1)
Known Positive	61 (64.2)	19 (100)	5 (100)
Wildtype virus	69(93.2)	5(6.8)	0
Detectable resistance n(%)	26 (27.4)	14 (73.7)	5 (100)
NNRTI only n(%)	23 (24.2)	2 (10.5)	4 (80.0)
NRTI only n(%)	0	1 (5.3)	0
NNRTI+NRTI n(%)	3 (3.1)	9 (47.4)	1 (20.0)
NNRTI+NRTI+PI n(%)	0	2 (10.5)	0
Phylogenetic clusters	1	0	0

Table 4 shows all resistance mutations by self-reported treatment. Amongst treatment naïve FSWs, 20 had the NNRTI mutations K103N, 4 had the K101E or V106E mutations. No PI mutations were seen in this group. For those on treatment (n = 14), 7 had the K103N mutations and 5 had the V106M or G190A mutations, and 2 and the K101E mutations. Amongst treatment defaulters (n = 5), 4 had the K103N mutation and 1 had the V106M mutation. The NRTI mutation M184V was also found in 1 participant. Overall, the number of FSWs with the K103N mutation was 68.9% (31/45), the K101E mutation was found in 6/45 (13.3%), the V106M mutation in 10/45 (22.2%), G190A in 5/45 (11.1%), and the NRTI mutation M184V was present in 13/45 (28.9%) of SWs.

**Table 4:** Treatment status by HIVDR mutations amongst FSWs with detectable DR in Soweto (n = 45), showing n (%).

	HIVDR Mutation	Naïve (n = 26)	On treatment (n = 14)	Defaulting(n = 5)	Total (n = 45)
NNRTI MUTATIONS	K103N (n = 31)	**20(76.9)**	7(50.0)	4(80.0)	31(69.0)
	V106M (n = 10)	**4(20.0)**	5(35.7)	1(20.0)	10(22.2)
	P225H (n = 4)	**-**	7(50.0)	1(20.0)	8(17.8)
	Y181C (n = 1)	**-**	1(7.1)	-	1(2.0)
	G190A (n = 5)	**-**	5(35.7)	-	5(11.1)
	K101E (n = 6)	**4(20.0)**	2(14.2)	-	6(13.3)
	Y188L (n = 1)	**-**	1(7.1)	-	1(2.0)
	Y181C (n = 1)	**-**	1(7.1)	-	1(2.0)
	G190A (n = 5)	**-**	5(35.7)	-	5(11.1)
	V179D (n = 2)	**1(5.0)**	1(7.1)	-	2(4.4)
	K101P (n = 1)	**1(5.0)**	-	-	1(2.0)
NRTI MUTATIONS	M184V (n = 13)	**-**	12(85.7)	1(20.0)	13(29.0)
	T215F (n = 1)	**-**	1(7.1)	-	1(2.0)
	K65R (n = 4)	**1(5.0)**	3(21.4)	-	4(8.9)
	K70R (n = 1)	**-**	1(7.1)	-	1(2.0)
	K70E (n = 2)	**-**	2(14.2)	-	2(4.4)
	D67N (n = 2)	**1(5.0)**	1(7.1)	-	2(4.4)
	M41L (n = 1)	**-**	1(7.1)	-	1(2.0)
	V75MI (n = 2)	**-**	2(14.2)	-	2(4.4)
PI	L90M (n = 2)	**-**	2(14.2)	-	2(4.4)

## Discussion

This cross-sectional study examined HIV-1 drug resistance amongst FSWs in Soweto who had unsuppressed viral loads. Disturbingly we found that 1:4 FSWs who were treatment naïve had TDR. This highlights the vulnerability of FSWs in Soweto to the sexual acquisition of HIVDR, and the resulting impact upon both treatment options and long-term survival. Additionally, more than a third of FSWs who were not virally suppressed had HIVDR (45/119), and three quarters of those on treatment but failing to suppress had HIVDR (14/19). This suggests the importance of providing services to SWs that they can actively engage in, and it highlights the importance of proactive monitoring of VL within sex work programs to ensure that timeous decisions are made in response to treatment failure. Clinical settings that provide services to SW may also be an environment to expand existing highly centralized VL testing services with technology capable of polyvalent testing (HIV VL, HPV and TB as example) that can be performed at the point of care. Such technology is becoming available such as the GeneXpert (Cepheid, Sunnyvale, CA) platform [32]. Furthermore, only mutations relating to first line regimens were found amongst treatment naïve SWs, highlighting the possibility that a quarter of FSWs will fail first line therapies for reasons unrelated to adherence. Our data highlights that most FSWs who are on treatment are adherent and virally suppressed. Findings also suggested that the VL of those who had HIVDR mutations was high, thus potentially resulting in the onward transmission of HIVDR in cases where mutations are more robust.

Our findings highlight the potential for sexually transmitted HIVDR. While, previous research has measured transmitted HIVDR at between 9–14% [9, 10], we found that more than one quarter (27,4%) of FSWs who were not virally suppressed and who reported being treatment naïve had HIVDR mutations. Research has previously shown that men who purchase sex are more likely to be HIV positive [17], not use condoms and have been found to be more violent when compared to men who do not purchase sex [18, 19, 33]. These partner behaviors in combination with the findings of this study highlight the increasing vulnerability of FSWs to HIV. Interventions which target partners of SWs are urgently needed to address both sexual risk behaviors and treatment adherence. Research is urgently needed to understand the prevalence of HIVDR amongst partners of FSWs and their transmission dynamics.

We found that the majority of FSWs in our study and who were on treatment (118/269), were virally suppressed (99/141). Extrapolating from this, we suggest that once on treatment, FSWs are likely to be adherent to their ART regimens. However, our data also shows that more than 75% of FSWs on treatment who are not virally suppressed have compromise to their first line regimens because of HIVDR. These FSWs ideally need to be managed according to the national protocol for virologic failure, which may result in being switched to a second line regimen. Given our previous findings regarding TDR, it is also possible that some of these failures are due to the sexual acquisition of HIVDR and not non-adherence.

Amongst those who are on treatment or defaulting treatment, it is important to note that several factors limited the ability of FSW to be 100% ART compliant. Research has shown that SWs are exposed to high levels of violence [13, 34], and that violence impacts women’s ability to be ART adherent [20]. Additionally, policing officials have been documented withholding access to ART for SWs in custody [35]. In addition, the population is particularly vulnerable to being displaced from the healthcare system. These factors are particularly critical in ensuring effective adherence to ART. We found that almost 75% of the sample who reported being on treatment but were failing to suppress had drug-resistant mutations–the most likely scenario is that these mutations represent acquired resistance, and it suggests that treatment interruptions have likely been experienced by these individuals. Given the complexities of their lives, many of these interruptions will likely be beyond the individual level of control and require renewed efforts to address the various structural factors which serve to drive the epidemic and SWs inability to effectively engage with health systems. Another finding among those on treatment with an unsuppressed viral load is that wildtype virus was identified upon genotypic testing in 26,3% of them (5/19). This illustrates another recognized use of drug resistance testing–the presence of wildtype virus in these individuals suggests non-adherence to treatment as the cause of the virologic failure [6]. This can avoid prematurely switching to a costly and burdensome second line regimen when the virus may still have been susceptible to the original one.

Drug resistance as a percentage of the total number of HIV positive participants in our study was higher than has previously been found amongst a SW cohort in Kenya (16.7% vs. 8.3%) [22]. Our findings were within a similar range to those from two Argentinian FSW studies (18.8–19%)[23, 24]. However, our study sample included individuals who were self-report treatment adherent as well as treatment naïve and treatment defaulters, whereas previous studies included only individuals on ART (verified and self-report, respectively). While modeling estimates suggest that in certain scenarios outlined previously HIVDR will be prevalent amongst 32% of the HIV positive population by 2032[9], our findings suggest that HIVDR may be increasing to this point as it is already at 16% amongst the Soweto SW population and that further research is urgently required to better understand this.

The finding that there was 1 phylogenetic cluster amongst treatment naïve FSWs adds to our concern in that it suggests a common source to their infection, who likely has multiple other partners, is inconsistent in condom use, [17–19] and may transmit HIVDR to other persons. In our study, less than half of FSWs had initiated ART and were currently on treatment, which was higher than in the SAHMS (45.6% versus 12.2%) [13]. This suggests that there are fundamental differences between FSWs in Soweto [15] from the overall metropolitan of Johannesburg [13]. This could it part be explained by the varying profiles of FSW between the township [15]. Similarly, to the SAHMS, we found that almost all FSWs in our study had previously tested for HIV, with < 20% of all HIV positive FSWs being unknown positives, and of these, less than one third had unsuppressed viral loads. This suggests that FSWs in Soweto are testing for HIV and that there is the potential for linkage to care to initiate and monitor their response to treatment. Individuals with unsuppressed viral loads were also more likely to have a lower CD4 count than those who were virally suppressed, suggesting poorer health outcomes for a subset of our sample group. When considering the WHO 90-90-90 targets we see a mixed picture. The proportion of HIV positive participants who knew their own status was encouragingly high, xx%, approaching the 90% target. However, as shown and discussed the other two WHO targets are far from being met, with only 43,9% already on ART treatment, and 70,2% of those on treatment who are virally suppressed. This of course impacted by the high number of drug resistant mutations in those who are in virologic failure.

This study had several limitations. Firstly, the sample size was small and does not allow for generalizability of findings. Secondly, treatment status was self-report and may not be an exact reflection of lifetime exposure to ART, especially for women who had received PMTCT (although this was probed during the survey), and no data was collected on specific regiments currently or historically used. Unsuppressed viral loads were defined as VL ≥500 copies/ml and not VL ≥400 copies/ml as in previous studies, thus 2 samples were excluded from HIVDR testing.

Our study suggests there may be alarming rates of HIVDR being transmitted between clients or intimate partners and FSWs in Soweto. This will potentially result in a large proportion of treatment naïve FSW having compromised first line therapies. Given the high HIV prevalence in this population and their vulnerability to violence and other health concerns[13, 15], it is critical that we begin to understand transmission dynamics and the outcomes of TDR. In addition, we found that three quarters of FSWs failing treatment had likely developed ADR. This suggests the frequency of treatment interruptions in this population which needs to be better understood for intervention development. Further, routine surveillance of VL must be rigorously undertaken amongst such high-risk populations to ensure that failure to suppress is proactively managed and second line therapies implemented timeously. Given the scale up of the *South African National Sex Worker HIV Plan 2016–2020[2]*, it is important that programmers are aware of the potential for first line resistance, and that more than one quarter of FSW initiated onto treatment and who are in virologic failure, may already have some form of resistance to first line. Furthermore, sex work programs in South Africa currently leverage nurse initiated ART for clinical service provision. Our data highlights the need for greater clinical oversight and understanding within these projects to ensure that resistance concerns are appropriately monitored and managed. Furthermore, routine HIVDR surveillance amongst key populations should become a priority within South Africa. This will ensure that healthcare officials, policy makers and program implementers are able to monitor and rapidly respond as HIVDR continues to show increasing trends amongst this population.

## Supporting information

S1 File Latex file for public release of data. (TEX)
